# Physical activity's impact on rural older adult health: The multiple mediating effects of education, income, and psychological capital

**DOI:** 10.3389/fpubh.2023.1173217

**Published:** 2023-04-17

**Authors:** Yujin Sun

**Affiliations:** School of Management, Suzhou University, Suzhou, China

**Keywords:** physical activity, older rural adults, psychological capital, multiple mediating, health

## Abstract

**Introduction:**

This study aims to explore the influence mechanism of rural older adult health. By examining the mediating roles of education, income, and psychological capital in physical activity's impact on health, this study provides a reference for lifestyle interventions to improve the health level of rural older adults.

**Methods:**

The analysis was conducted on a sample of 1778 rural older adults from CGSS2017, and data were analyzed using PROCESS V4.2 for multiple mediating effects.

**Results:**

The findings indicate that physical activity impacts rural older adult health through multiple mediating pathways. The mediating role includes seven paths, comprising the independent effects of three mediating variables of income, education, and psychological capital, and the chain mediating effects generated together.

**Discussion:**

Based on the influence mechanism of health on rural older adults, optimizing policy focus and developing a precise, interconnected, and sustainable health security system for older adults is necessary. These research results are of practical significance for advancing healthy aging in rural areas.

## 1. Introduction

Public health issues have become increasingly important to society, with health level a crucial measure of success (1, 2). Age and group disparities exist in health status, with older adults being particularly vulnerable (3). Health status is a significant factor that influences the life quality of older adults (4). Healthy aging is essential for economic and social development and is the foundation for formulating various social public policies (5). While life expectancy is increasing, the period of illness is disproportionately expanding (6), and chronic diseases in older adults are a significant health concern (7). Older adults require health care and nursing services, with rural older adults particularly needing attention (8, 9).

The issue of rural aging is a major global challenge (10). The diversity of rural areas is an essential consideration in rural aging (11), where, health risk factors and social and economic factors can influence healthy aging in rural areas (12). Changes in the economic structure have reduced rural families' dependence on the land. In contrast, changes in intergenerational relationships have led to declining traditional values and a lack of appropriate social security systems for rural older adults (13). As a result, rural older adults are a particularly fragile group (14) with high levels of disability (15) and caregiving challenges (16), and about one-third of rural older adults experience depressive symptoms (17). Rural older adults are also at risk of social isolation (18), experiencing social disconnectedness (19). In the context of population aging, exploring the mechanism of health influence on rural older adults can provide for policymaking to improve their health level.

Health is the ability to adapt and self-manage, encompassing physical and mental health (20). It is an essential capability, and all individual activities are based on it (21). Health has personal and public attributes and is the foundation for accumulating human capital (22). Health status results from continuous accumulation throughout life and will decline with age (23). The incidence of health problems varies at different life cycle stages (24). Relative economic backwardness, inadequate medical resources, and lagging health security systems in rural areas have resulted in a long-term accumulation of health disadvantages for older rural adults throughout their lifecycles (25). As a vulnerable group, they are more likely to encounter health risks (26). Cancer incidence and mortality rates among rural residents are higher than those in urban areas (27), and improving the health of older rural adults is vital to healthy aging (28).

Lifestyle, personal, and social factors impact the health of older rural adults (17, 18). Previous research has shown that regarding lifestyle, physical activity, social interaction (29), and diet (30) affect the health of older rural adults. Regarding personal factors, gender (31), income (32), education (33), and marital status (34) all affect the health of older rural adults. Living arrangements are a personal choice for older rural adults (35). They prefer to stay at home and in the community (36), with aging in place (37). Spouses can provide daily care and emotional support (34), and living arrangements and support from children can affect the health of older rural adults (38). Regarding social factors, healthcare needs in rural areas are not being met (39), and healthcare practices affect the health of older rural adults (40, 41). Community resources, infrastructure (42), internet use, and the digital divide (43) affect depression levels in older rural adults. Environmental factors (44) and pollution (45) also affect the health of older rural adults. Health influencing factors result from the interaction and accumulation of multiple factors, which can affect sustainable livelihoods and improve the quality of life for older rural adults (46). Therefore, this study aims to investigate the specific mechanism of rural older adult health.

## 2. Literature review

*Physical activity* significantly impacts health (47, 48). It is part of a healthy lifestyle (49, 50), and WHO believes physical activity can improve all aspects of health and provide multiple benefits (51). Physical activity effectively augments health beyond pharmaceutical treatments, lowering disease incidence, improving quality of life, and increasing healthy lifespan (52, 53). Sedentary behavior is a risk factor for older adult health (54) and contributes to overall mortality risk (55). Increasing physical *activity* is an essential strategy for preventing chronic disease (56) and can reverse its effects (57). A linear relationship exists between physical activity and health status (58). Physical activity is a health-promoting behavior (59). Lack of exercise leads to health risks (60, 61). Appropriate physical activity can improve health levels and reduce disability risk in older adults (15, 62), helping to alleviate the trend of disability in older adults (63). Physical activity can prevent frailty (64), delay the development of frailty (65), relieve depression, reduce anxiety, and improve mental health. These outcomes have been observed in residents of different countries (66, 67). Moreover, physical activity strengthens older adults' cognitive function (68). It is a low-cost and effective cognitive function intervention (47, 69). Previous literature has established the impact of physical activity on health. This study primarily concerns how physical activity affects older rural adult health.

Education affects health, providing positive returns on investment (70). The theory of acquired effectiveness states that education is the most critical factor affecting individual health. It endows individuals with various resources, enhancing their sense of control over life and promoting a healthy lifestyle (71). Education and health are both human capital and are mutually reinforcing (72). According to the cumulative advantage and disadvantage hypothesis, the advantages of factors such as education will gradually strengthen. Health differences will continue to expand as health results from accumulated social capital and experience (73). Acceptance and decisions of health risks by older rural adults relate to their level of education, and the level of education has a significant positive effect on the health of older rural adults (33).

Income affects health (74). It is a core indicator of social and economic status, with higher income leading to more robust investment in health capabilities. Income inequality is the main factor of population health differences, and increasing the income of vulnerable groups can reduce health inequality and improve health levels (75). In rural areas, family pension functions are magnified, following the labor lifestyle of living and working into old age and continuously bringing income to the family (6). The increase in income positively affects health as a family's economic support (76). A significant relationship exists between income inequality and health (77). The pension level of older rural adults is low, and pension affects health (32). Medical expenditure is the primary source of the vulnerability of individual and family economic statuses (24), and poverty has a detrimental effect on the health of older rural adults (78).

*Psychological capital* (PsyCap) affects health (79). It is a core psychological resource, an individual's positive psychological state, including hope, efficacy, resilience, and optimism (80), with developability (81). As a personal resource, psychological capital affects mental health over time (82). It is related to positive emotions, guiding individuals to produce positive behaviors (83), and psychological capital affects successful aging (84). According to the expansion and construction theory of positive emotions, individuals with high psychological capital have more flexible cognitive and behavioral models. They are more likely to obtain energy from the outside when facing external risks (85). Positive psychological capital can protect individuals' health from harm in adversity (86). Psychological capital can improve health levels and alleviate depression in older rural adults (79, 87).

*Health* is a complex and comprehensive issue that requires a comprehensive measurement approach. Healthy aging emphasizes the centrality of functional ability, which encompasses personal capabilities, environmental features, and their combination (5). In the functional-centered healthy aging paradigm, physical activity and psychological capital reflect personal capabilities, while education and income are environmental features (88). Previous studies have identified physical activity, income, education, and psychological capital as predictors of health. However, how these variables interact with each other to influence health has not been thoroughly analyzed, and the underlying mechanisms are unclear.

Based on the findings of the studies described above concerning possible predictors of the health of older rural adults, this study examines the following hypotheses:

Hypothesis 1: Education plays a mediating role in the relationship between physical activity and health.
Hypothesis 2: Income plays a mediating role in the relationship between physical activity and health.
Hypothesis 3: Psychological capital plays a mediating role in the relationship between physical activity and health.
Hypothesis 4: Education, income, and psychological capital have multiple mediating effects between physical activity and health.

This study uses income, education, and psychological capital as mediating variables to construct a multiple mediating effect model and test physical activity pathways affecting the health of older rural adults.

## 3. Methods

### 3.1. Data sources

This study utilized data from the 2017 China General Social Survey (CGSS), a publicly available data source collecting data at multiple levels, including individuals, households, communities, and society, since 2003. The CGSS is widely used to study social issues due to its high data quality (89), strong applicability, and representativeness (90). After cleaning the original data, urban residents were excluded, followed by individuals below 60, resulting in a sample of older rural adults 60 and above. After removing missing and invalid samples, 1,778 valid samples were obtained, covering 26 provinces and cities in China and comprising 920 males and 858 females. The data contains comprehensive information on the individual characteristics and family situations of older adult respondents, providing robust data support for studying the relationship between physical activity and the health of older rural adults.

### 3.2. Measurements

The dependent variable was health, measured by physical and mental health (1). The question “How do you feel about your current physical health?” (70) assessed physical health. The re-coded question “How often do you feel depressed or discouraged?” (90) assessed mental health, with higher scores indicating better health. The independent variable was physical activity, measured by the question, “In the past year, do you often participate in physical activity during your leisure time?” (91). Answers were re-coded as *never* or *rarely* (0) and *daily* or *several times a week* (1).

Education, income, and psychological capital were the mediating variables. Education was measured by educational attainment (70). Income was measured by household economic status (91, 92). Psychological capital has four primary positive psychological resources, assessed by the indicators in the survey questionnaire (80, 93).

The hope dimension was measured by the questions “When things are uncertain, I usually expect things to turn out for the best” and “Overall, I expect more good things to happen to me than bad things.” The efficacy dimension was measured by “I think I am quite successful now.” The resilience dimension was measured by the questions “I am currently doing my best to pursue my goals” and “There are many solutions to the problems I am facing now.” Finally, the optimism dimension was measured by the questions “I have a positive attitude toward my future” and “I often get upset about small things.” After re-coding the reverse questions and summing up the scores, the higher the composite variable, the better the psychological capital. The four-dimensional psychological capital evaluation index had a Cronbach's α of 0.719, indicating a high level of reliability.

Based on previous studies, control variables included gender (31), marital status (34), and living arrangements (16, 94), all of which were represented as dummy variables.

### 3.3. Statistical analysis

Data were analyzed using PROCESS V4.2 for multiple mediating effects, which improved the accuracy of the estimation compared to the ordinary mediating effect model (95). The mediating effects were tested using the bootstrap method with 5,000 repeated sampling.

## 4. Results

### 4.1. Descriptive statistics and correlation analysis

The descriptive statistics and correlation analysis results of the study variables are shown in Table 1. The Spearman correlation coefficient revealed that physical activity, education, income, and psychological capital were significantly positively correlated with health.

**Table 1:** Correlation matrix of main variables.

	**M**	**SD**	**1**	**2**	**3**	**4**	**5**
1 Physical activity	0.22	0.41	–				
2 Education	1.91	0.86	0.166^**^	–			
3 Income	2.28	0.80	0.183^**^	0.183^**^	–		
4 PsyCap	25.99	5.54	0.205^**^	0.267^**^	0.301^**^	–	
5 Health	6.22	1.78	0.150^**^	0.215^**^	0.302^**^	0.426^**^	–

** P < 0.01.

### 4.2. Regression analysis

The results of the regression are presented in Table 2. Physical activity significantly positively predicted education (β = 0.244, *p* < 0.05), income (β =0.369, *p* < 0.01), and PsyCap (β = 0.354, *p* < 0.01). When physical activity, education, income, and PsyCap were entered into the regression equation simultaneously, physical activity (β = 0.216, *p* < 0.05), education (β = 0.118, *p* < 0.01), income (β = 0.148, *p* < 0.01), and PsyCap (β = 0.331, *p* < 0.01) significantly predicted the health of older rural adults.

**Table 2:** Regression analysis among variables.

**Regression equation**		**Overall Model Fit**			**Significance**	
**Outcome variable**	**Predictor variable**	***R***	***R** ^2^*	***F***	β	***t***
Education	Physical activity	0.394	0.155	22.367^**^	0.244	2.367^*^
Income	Physical activity	0.227	0.051	5.276^**^	0.369	3.354^**^
	Education				0.141	2.928^**^
PsyCap	Physical activity	0.384	0.148	13.987^**^	0.354	3.352^**^
	Education				0.163	3.542^**^
	Income				0.225	5.217^**^
Health	Physical activity	0.482	0.232	20.887^**^	0.216	2.128^*^
	Education				0.118	2.670^**^
	Income				0.148	3.525^**^
	PsyCap				0.331	7.677^**^

* P < 0.05,

** P < 0.01.

### 4.3. Mechanism analysis

**Figure 1:**
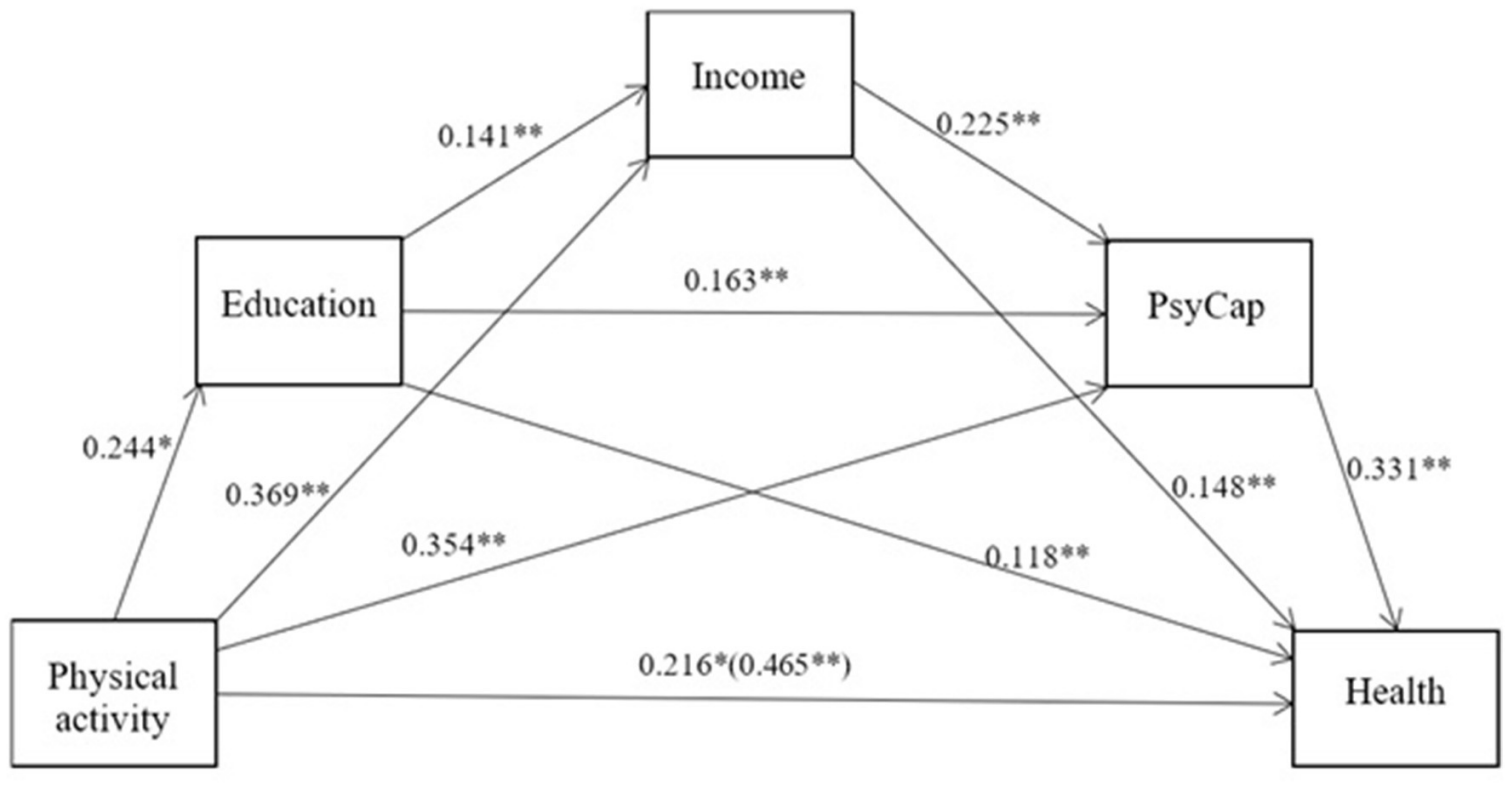
Chain mediating model of physical activity, education, income, and PsyCap. ^*^P < 0.05, ^**^P < 0.01.

Physical activity significantly predicted health (β = 0.465, *p* < 0.01). A chained mediating effect test was conducted using PROCESS, with older rural adults' health as the dependent variable and physical activity as the independent variable, incorporating mediating and control variables. Figure 1 depicts the results. Education, income, and psychological capital play a chained mediating role between physical activity and health.

Table 3 shows the confidence intervals of the indirect effects, found to be statistically significant using the Bootstrap method. Enhancing physical activity, education, income, and psychological capital can effectively improve the health level of older rural adults. The results indicated that the indirect effect was 0.029 in path 1 with education as the mediator, 0.055 in path 2 with income as the mediator, 0.117 in path 3 with psychological capital as the mediator, 0.005 in path 4 with education and income as the mediators, 0.013 in path 5 with education and psychological capital as the mediators, 0.027 in path 6 with income and psychological capital as the mediators, and 0.003 in path with education, income, and psychological capital as the mediators. Hypotheses 1, 2, and 3 have been confirmed.

**Table 3:** The results of the mediating effect.

	**Effect**	**BootSE**	**BootLLCI**	**BootULCI**	**Relative Effect**
Indirect effect	0.249	0.054	0.149	0.361	53.55%
Path 1	0.029	0.018	0.000	0.072	6.24%
Path 2	0.055	0.024	0.017	0.107	11.83%
Path 3	0.117	0.042	0.040	0.208	25.16%
Path 4	0.005	0.004	0.000	0.014	1.08%
Path 5	0.013	0.008	0.001	0.031	2.80%
Path 6	0.027	0.011	0.011	0.052	5.81%
Path 7	0.003	0.002	0.000	0.007	0.65%

The mediating effect test results demonstrated that physical activity influences health through multiple mediations of education, income, and psychological capital. Therefore, Hypothesis 4 has also been validated. With a total mediating effect of 0.249, they account for 53.55% of the total effect of physical activity on the health of older rural adults.

## 5. Discussion

Enhancing the health level of older rural adults requires identifying the mechanism of influence. Research findings indicate that education, income, and psychological capital are the mechanisms by which physical activity impacts the health of older rural adults. Physical activity can improve overall health in older adults and has significant benefits (96). Moreover, physical activity is positively related to and is critical in achieving healthy aging (97). It can be initiated from an early life cycle stage, with robust plasticity, lower cost, and more significant benefit in intervening against and preventing health risks. Intervening with physical activity for older adults is a cost-effective way to improve their health (98). Enhancing physical fitness can lead to lifelong health benefits, enhance the quality of life of older rural adults, and result in sustained health benefits (48, 99, 100).

### 5.1. Independent mediating effects

Previous studies have found a relationship between physical activity and education, and education has been linked to health (71, 101). Physical activity and education are forms of cultural capital that positively influence rural resident health (92), thus supporting the findings of a mediating effect study of these variables. While some studies have suggested a link between education and physical activity (102–104), identifying causal relationships requires specific research contexts and a constant conjunction of variables (105). In this study, from a physical activity perspective, education, income, and psychological capital as mediators can help to understand the relationship between physical activity and health (95). In addition, physical activity has been identified as an effective way for individuals to increase their income (91).

Furthermore, physical activity can improve health by enhancing economic status (106). Personal wealth has been found to affect health (107). It has been suggested that the fewer public products and services provided by society, the more significant personal income is to health (108), consistent with the mediating effects of physical activity-income-health. Physical activity has been found to predict psychological capital positively (109). In addition, physical activity may foster and improve psychological capital (81, 110), which is related to health. The theory of planned behavior suggests that behavior beliefs affect attitude formation (111). Older rural adults believe that physical activity will affect health and build psychological capital, consistent with the mediating effects of physical activity-PsyCap-health.

### 5.2. Multiple mediating effects

Physical activity has been identified as a preventive health behavior (112), which can be explained by the Health Belief Model (113). It has been found that there is a positive correlation between physical activity and educational level (71), as individuals accumulate human capital through education, which can help them to increase their income (114). Income is economic capital that provides access to more health resources (92). Previous studies have shown that physical activity affects education and income (115). Education level and income are two indicators of social and economic status (116), and it has been observed that older adults' health is positively associated with these factors (117). Furthermore, research has shown that education affects health through income (118), which aligns with the mediating effects of physical activity-education-income-health.

Physical activity is linked to educational level. Individuals having higher educational levels exhibit a better social mentality, higher cognitive level, and more optimistic attitudes (71, 101). The Human Capital Theory suggests that education can affect health through psychological resources (119). Education is a dividing line for health. Individuals shape psychological capital through the educational process, which promotes health behavior intentions to reduce risks (120), thus explaining the mediating effect of physical activity-education-PsyCap-health.

Previous studies have demonstrated that physical activity can bring more income to individuals, with an income effect (121, 122), and income can improve social and economic status (91). Physical activity is cultural, and income is economic capital. Moreover, the durable elements of psychological capital constitute social capital (123). All of these forms of capital are integrated within individuals, mutually influencing and interacting with each other. Health is human capital, affected by cultural, economic, and social capital (79), consistent with the mediating effects of physical activity-income-PsyCap-health.

The Grossman health demand model can explain the multiple mediating model path constructed. This model postulates that health is a durable capital stock (124) with dual attributes of consumption and investment (22). Individual health stock decreases with age. However, it can be augmented through investment (124). Physical activity and education are considered health investment behaviors. Education influences resources and determines the efficiency of health investment. People with higher educational levels tend to have larger incomes, which can be used to purchase wellness services and invest in health capital. Moreover, education affects livelihood resilience (125), forming positive psychological capital in increasing investment and affecting older rural adults' health. The research results confirm the Grossman model.

### 5.3. Implications

When confronted with unpredictable future health-related risks (126), developing coping strategies and institutional safeguards to optimize policy focus and ensure the social effectiveness of policies is required (70). In addition, an age-friendly, sustainable health security system for older rural adults should be established to promote healthy aging in rural areas (127).

First, when constructing a multi-dimensional health security system for older rural adults, factors such as education, physical activity, and psychological capital should be considered to bring universal health benefits to older rural adults. Particular attention should be paid to older rural adults with low income, low educational levels, and low psychological capital. In addition, precise identification mechanisms for vulnerable groups should be established to ensure they can enjoy policy benefits.

Second, health education is crucial. Education transmits health concepts and prevention information and actively guides older rural adults to engage in physical activity. It encourages effective health decision-making, cultivating healthy lifestyles and health literacy (128), and reducing risks.

Third, creating public service facilities suitable for physical activity and considering the needs of older adults with an inclusive approach is essential. These facilities will provide quality public services, upgrade educational levels and expand income sources for older adults. Furthermore, they will refine social security systems and bolster the psychological capital of older rural adults.

### 5.4. Limitations and future research

Compared with previous studies, this paper makes several possible contributions. First, based on verifying the effect of physical activity on health using CGSS data, this paper uses income, education, and psychological capital as transmission paths. It uses mediating effect model to study how physical activity impacts the health of older rural adults. Second, prior literature has largely overlooked psychological capital's role in the association between physical activity and health. This paper employs psychological capital as a mediating factor to investigate the health of older rural adults. Third, by leveraging multiple mediating effects to elucidate the health of older rural adults, seven pathways were identified, thus augmenting the existing empirical research.

The core value of this research lies in investigating the effect of physical activity on the health of older rural adults and the mediating roles of education, income, and psychological capital. However, this study has some limitations. First, the health of older rural adults is affected by numerous factors. A considerable time lag may occur, and the effects are cumulative. Thus, only physical activity's influence on health has been studied, which cannot fully explain the long-term dynamic evolution of older rural adults' health. This area requires further exploration.

Regarding influence mechanisms, only education, income, and psychological capital are discussed, which overlooks other aspects. Future research should explore other mechanisms to supplement this paper's findings. Finally, research on older rural adult health should be expanded. More extensive research will help to balance instrumental and value rationality and benefit older rural adults' health and well-being.

## 6. Conclusions

Health is a universal desire and necessity for human beings. It serves as significant human capital, promotes individual capability, and is an essential resource for society. This study, from the perspective of income, education, and psychological capital, examines the influence mechanism of physical activity on older rural adult health. The findings suggest that income, education, and psychological capital are mediating variables for physical activity to affect older rural adults' health. Physical activity affects the health of older rural adults through multiple mediating effects of income, education, and psychological capital. This mediation effect includes seven paths, including the independent effect of the mediating variable and the chain mediating effect generated together. In light of the mechanism of health influence on older rural adults, this research optimizes the policy focus and constructs a precise, interconnected, and sustainable health security system. The research results are of practical significance for advancing healthy aging in rural areas.

## Data availability statement

The datasets presented in this study can be found in online repositories. The names of the repository/repositories and accession number(s) can be found at: http://cgss.ruc.edu.cn/index.htm.

## Author contributions

YS designed the study, performed the statistical analysis, wrote the first draft, polished the manuscript, and approved the submitted version.
